# Supplementary material for: The causal relationship between the human gut microbiota and pyogenic arthritis: a Mendelian randomization study
Source: Front Cell Infect Microbiol. 2024 Nov 26;14:1452480. doi: 10.3389/fcimb.2024.1452480 (PMC11629706; doi:10.3389/fcimb.2024.1452480)
Supplement: Supplementary file 1 [file DataSheet1.zip › mendelian test/ebi-a-GCST90027648.csv_leave_one_out.pdf]

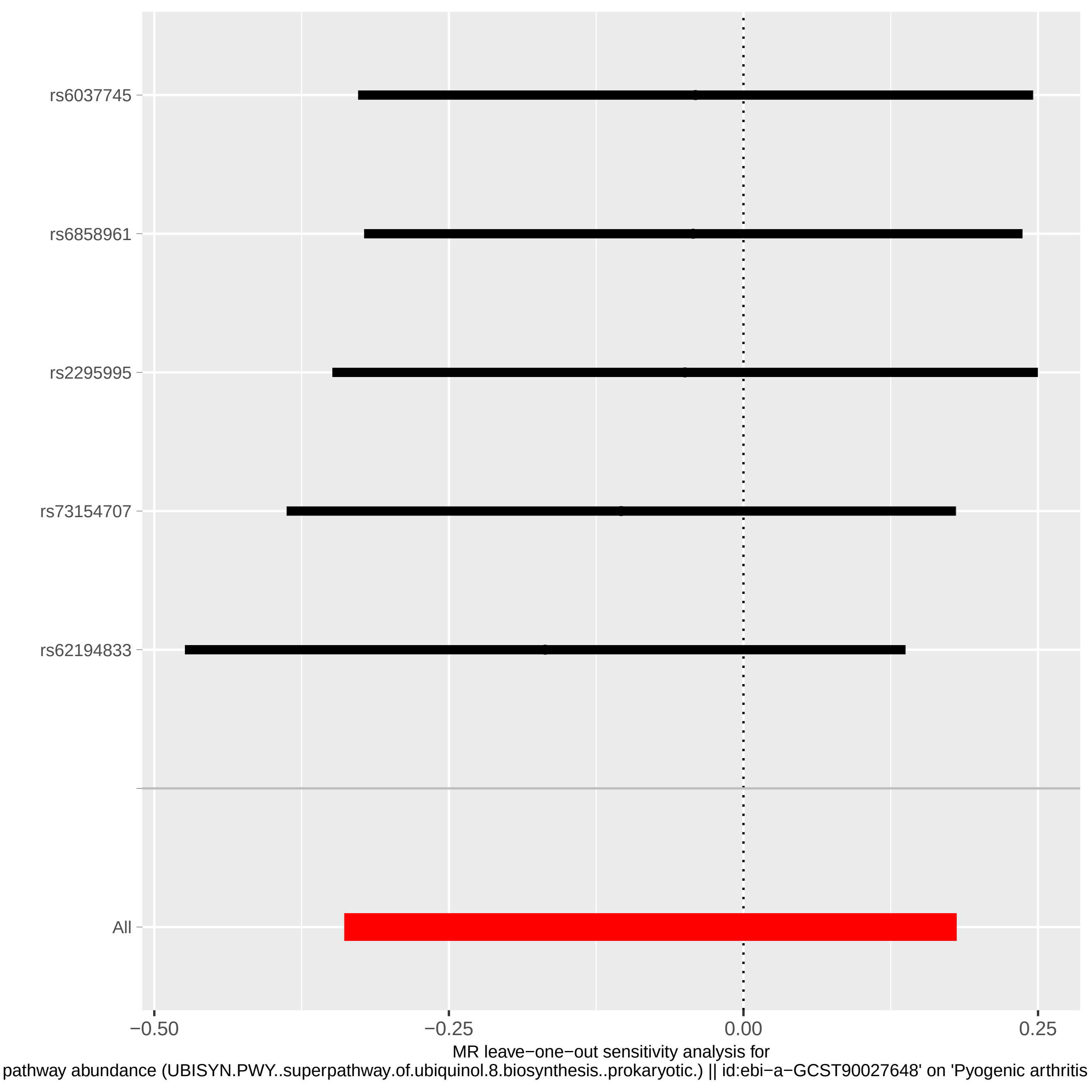

rs6037745
rs6858961
rs2295995
rs73154707
rs62194833
All
−0.50
−0.25
0.00
0.25
MR leave−one−out sensitivity analysis for
pathway abundance (UBISYN.PWY..superpathway.of.ubiquinol.8.biosynthesis..prokaryotic.) || id:ebi−a−GCST90027648' on 'Pyogenic arthritis
